# Trends and patterns of sexual assaults in Lagos south-western Nigeria

**DOI:** 10.11604/pamj.2016.24.261.9172

**Published:** 2016-07-20

**Authors:** Oliver Chukwujekwu Ezechi, Zaidat Adesolamusa, Agatha Nkiru David, Agatha Eileen Wapmuk, Titilola Abike Gbajabiamila, Ifeoma Eugeniaidigbe, Paschal Mbanefo Ezeobi, Aigbe Greg Ohihoin, Innocent Achanya Otobo Ujah

**Affiliations:** 1 Clinical Sciences Division, Nigerian Institute of Medical Research Yaba Lagos, Nigeria

**Keywords:** Sexual assault, sexual violence, rape, women, Nigeria

## Abstract

**Introduction:**

Sexual assault is a severely traumatic experience that disproportionally affects women and girls. Yet there is limited information on the subject in our environment. This study was conducted to determine the trend and pattern of sexual assault among Nigerians.

**Methods:**

A retrospective study of sexual assault victims managed at a large clinic in south west Nigeria. Victims were identified from the programme data base and case files retrieved from medical records department. Relevant information was extracted and managed with SPSS for windows version 19.

**Results:**

Steady increase in the proportion of reported cases of sexual violence over the years (P < 0.0001) was observed. Sexual assaults were recorded among the males (6.1%), although female victims were in the majority (93.9%). Sexual assault was found to be higher in person’s <20 years and the unmarried. Most sexual assault occurred during the day time. Assailants were mostly persons known to the victim (52.0%) and the assault occurred mostly in the assailants’ house or office (48.5%). Sexual assault through vaginal route only (87.2%) was the most common route of sexual assault. Threat of violence (31.1%) and physical force (29.6%) was the common methods for overcoming the victims. Follow up was completed by 75.0% of the victims.

**Conclusion:**

Sexual assault is common in our environment, with increasing prevalence and change in pattern. Young persons aged less than 20 years constitutes the majority of victims and assailants were mostly persons known to them. The current public education on the evils of sexual violence should be intensified.

## Introduction

Sexual assault is a severely traumatic experience that disproportionally affects women and girls [1, 2]. It is often associated with psychological, physical, social distress and occasionally result in fatality either from shock, severe injury or murder by the perpetrator in an attempt to conceal his identity [3, 4]. Sexual assault encompasses a wide range of activities ranging from rape to physically less intrusive sexual contacts, whether attempted or completed [1, 3, 4]. It involves lack of consent; the use of physical force, coercion, deception or threat; and/or the involvement of a victim that is asleep, unconscious, under aged, mentally incapacitated or physically impaired as a result of voluntary or involuntary alcohol or drug consumption [1, 5]. Although penile vaginal penetration or attempted penetration is the commonest form of sexual assault, penile penetration or attempted penetration of the anus or mouth without consent is increasingly being reported [1]. Breast or genital fondling, forced or coerced touching of another’s genitalia and forced or coerced anal or vaginal penetration with other body parts or object are other forms of sexual assault [6]. Sexual assault is a pandemic crime that is characteristically underreported, more especially in the low income countries [4], because of the enduring culture of male dominance, female social and economic disempowerment and poor or non-prosecution of sex offenders [4, 7]. According to World Health Organization reports, one in every five women is a victim of sexual assault [8] and globally, 35% of women have experienced either physical and/or sexual intimate partner violence or non-partner sexual violence [9]. The reported figures are said to be inaccurate and often underestimate as most cases of sexual assault are under-reported by the victims because of the associated stigma [10]. In Nigeria, only 2 of 40 cases of rape are reported, attributing this amongst other reasons, to the arduous legal requirements needed to prove the cases and the associated stigma [1, 11]. The reported incidence of sexual assault in Nigeria ranged from 13.8% among female students in Maiduguri [10] to 15% among young females in Ibadan [12]. Until 2002, the management of sexual assault especially prevention was a major challenge in our setting [1, 10]. The introduction of the Federal Government of Nigeria antiretroviral drug access programme in 2002 changed this poor outlook as, a number of post exposure prophylaxis programmes was established across the country. Public health education and campaign on the need for persons occupationally and non-occupationally exposed to HIV and other infectious agents to avail themselves to these services inorder to avert infection were intensified. It is hoped that a lot more persons sexually assaulted will avail themselves of this preventive service even if they do not want to report to the police. This will hopefully assist in narrowing the gaps in the reported cases of sexual assault in Nigeria and worldwide. This study was conducted to determine the trend and pattern of sexual assault on women, girls and men among Nigerians who presented for HIV post exposure prophylaxis between July 2006 to June 2015 at the HIV treatment centre, Nigeria Institute of Medical Research Lagos.

## Methods

The study was conducted at the HIV treatment centre, Nigerian Institute of Medical Research, Lagos. The centre started operations in 2002 following the commencement of the Federal Government of Nigeria Antiretroviral access programme. The centre currently provides comprehensive HIV care, treatment and support for over 23,000 patients. Sixty five percent of the patients come from Lagos and the rest from the other 5 states of southwestern Nigeria as well as from north-central, south-south and south-eastern Nigeria. A little over 0.03% comes from neighbouring West African countries. Patients are enrolled into the HIV treatment programme following a referral from the HIV Counselling and Testing Centre, Nigerian Institute of Medical Research, Lagos or transfer from other Government of Nigeria HIV treatment centres. The HIV treatment centre has a HIV post exposure prophylaxis programme that operates a free 24 hour service. All cases of occupational and non-occupational exposure to infectious agents are provided services using a standardised management protocol. Patients are either self-referred or referred from public and private hospitals/clinics, NGOs and the police. Information was collected from the patients using a pre-assessment, entry and visit forms. Collected information was entered prospectively into the programme data base. All cases of sexual assault seen over a period of 10 years (January 2006 to December 2015) were retrieved from the programme data base, de-identified and analysed with SPSS for windows version 19.

### Compliance with ethical standards

**Funding:** This study was funded by research fund from Nigerian Institute of Medical Research Lagos. Treatment and care of the patients was supported by AIDS Prevention Initiative in Nigeria (APIN).

**Ethical approval:** All procedures performed in this study were in accordance with the ethical standards of the Institutional Review Board, Nigerian Institute of Medical Research, Lagos who gave the ethical approval for the study and with the 1964 Helsinki declaration and its later amendments or comparable ethical standards.

## Results

A total of twenty one thousand one hundred and ninety nine patients presented at the treatment centre for services including post exposure prophylaxis during the ten year period; of which one hundred and ninety six were cases of sexual assault (0.9%). Table 1 shows the trend in cases of sexual assault among patients seen at the treatment centre. There was a steady increase in the proportion of patients that presented for sexual assault among the patients. There was a tenfold increase in the proportion of patients presenting with sexual assault from 0.2% in 2006 to 2.0% in 2015 (Chi Square for trend 51.45; P < 0.0001). Table 2 shows the socio-demographic characteristics of sexual assaulted victims. The ages ranged from 3 to 53 years with a mean of 21.2 ± 8.8 years. Over two third of the patients (68.3%) were aged less than 30 years, with those aged 10-19 years constituting 35.2% of cases. Majority of the sexual assault victims were students (51.5%) and unmarried (73.9%). Of the 196 cases of sexual assault victims, while females were in the majority (184; 93.9%), the remaining 12(6.1%) patients were males. The age of the male victims ranged from 19 to 41 years with a mean of 24.9 ± 4.9 years. The type of sexual assault by sex of the victim is shown in Table 3. Sexual assault through vaginal route only was the commonest type of assault (171; 87.2%), followed by assault through anal penetration only (11; 5.6%). In the remaining thirteen cases, assault was through the mouth (4; 2.0%) and multiple routes (9; 4.6%). Among the twelve male victims, nine (75.0%) were assaulted through the anus. The time of sexual assault is shown in Table 4. While majority of the assaults occurred during the day (53.6%), in the remaining ninety one cases the assaults were during the night (46.4%). While over two-third (75.3%) of cases occurred during the day, in those aged above 30 years majority of the assault (75.6%) occurred at night (p = 0.000. OR: 5.7; 95% CI: 2.9-11.1). The time interval between assault and presentation in the centre range from about 4 hours to 96 hours; with majority presenting within 72 hours (90.8%). Only 34.6% of the patients presented within 24 hours (Table 4).

**Table 1:** Trends in cases of sexual assault during the 10 year study period (2006-2015)

Year of presentation	Number of patients presented for HIV services	Number of Sexual assault cases (%)
2006	2277	4(0.2)
2007	2868	8(0.3)
2008	2989	13(0.4)
2009	2657	18(0.7)
2010	2318	29(1.3)
2011	2327	35(1.5)
2012	1644	18(1.1)
2013	2211	30(1.4)
2014	1178	23(2.0)
2015	730	18(2.5)
	21,199	196(0.9)

**Chi Square for linear trend = 51.45; P value < 0.0001**

**Table 2:** Sociodemographic characteristics of the sexual assault patients

Characterisitics	Number of Patients (%)
Sex	
Male	12(6.1)
Female	184(93.9)
Age (Years)	
< 10	12(6.1)
10 - 19	69(35.2)
20 - 29	53(27.0)
30 - 39	45(23.0)
> 40	17(8.7)
Mean (SD)	21.2 ± 8.8 years
Occupation	
Pre-school	9(4.6)
Student	101(51.5)
Unemployed	9(4.6)
Apprentice	17(8.7)
Domestic servant	5(2.6)
Civil servant	13(6.6)
Artisan/ Petty trading	11(5.6)
Professionals	18(9.2)
Educational level completed	
None	16(8.2)
Primary	23(11.7)
Secondary	98(50.0)
Tertiary	59(30.1)
Marital status	
Married	141(71.9)
Not married	55(28.1)

**Table 3:** Route of sexual assault by sex

Route of sexual assault	Males n = 12 (6.1%)	Females n = 184 (93.9%)	Total n = 196 (100.0%)
Vaginal route only	-	171(92.9)	171(87.2)
Anal Route only	9(75.0)	2(1.1)	11(5.6)
Oral route only	3(25.0)	1(0.5)	4(2.0)
Vaginal and anal route	-	3(1.6)	3(1.5)
Vaginal and oral route	-	5(2.7)	5(2.6)
Vaginal, anal and oral routes	-	1(0.5)	1(0.5)

**Table 4:** Time of occurrence of sexual assault by age group

Age group	Day (%)	Night (%)	Total (%)
Less than 20	61(75.3)	20(24.7)	81(100)
20 – 29	28(52.8)	25(47.2)	53(100)
30 – 39	11(24.4)	34(75.6)	45(100)
Greater than 40	5(29.4)	12(70.6)	17(100)
	105(53.6%)	91(46.4%)	196(100.0)

While the place of assault in most cases were the assailant’s home or office (48.5%), in others the assault took place at victims home (19.9%), uncompleted building (10.7%), street corner (11.2%), friend’s house (5.1%), taxi/bus (3.1%) or hotel/guest house (2.6%). The assailants’ were known to the victims in majority (102; 52.0%) of cases (friends (27.0%), neighbour (17.9%), authority figure (4.6%) and relations (2.6%). The other assailants were unknown to the victim (52; 26.5%) or criminals/armed robbers (42; 21.4%). The number of assailants ranged from one to seven. While in the majority of cases only one assailant was involved in the assault (139;70.9%), in the remaining cases, two assailants (37;18.9%), three or more assailants (15;7.7%) and unknown number of assailants (5;2.5%) were involved in the attack. The method of overcoming the victim ranged from physical force (29.6%), threat of violence (31.1%), deceit (19.4%), drug/alcohol (17.3%) or money (2.6%). Fifty five (28.1%) out of the 196 victim sustained physical injuries (9.2%) and anogenital injuries (18.9%). Majority of the genital injuries were sustained by victims aged less than 20 years. All the patients had HIV screening and only one was confirmed HIV positive (0.5%). Only 67(34.2%) of the patients had hepatitis B screening. All the victims except 19(9.7%) received post exposure prophylaxis One hundred and seventy four women received emergency contraception (95.0%) and 147(75.0%) completed 3 month follow up. Only 21.9% of the victims had reported to the police before presentation.

## Discussion

The prevalence of sexual assault among patients presenting for various HIV services at our centre was 0.9%, with a steady increase over the years (P < 0.0001). Sexual assaults were recorded among the males (6.1%), although female victims were in the majority (93.9%). Sexual assault was found to be higher in teenagers and the unmarried. Most sexual assault occurred during the day time, however among older adult aged above 30 years majority of the assault were at night time. Assailants were known to the victim in majority of the cases and the assault occurred either in the assailants house/office (48.5%) or victims home (19.9%). Although majority (90.8%) of the victims presented within 72 hours when intervention could be effective, only 34.6% presented within 24 hours of assault. The assailants were known to the victims in majority (52.0%) of cases and were either a friend, neighbour, authority figure or a relation. Assaults were perpetrated by one person in majority of cases (70.9%). Sexual assaults through vaginal route only (87.2%) was the most common route of sexual assault. Threat of violence (31.1%) and physical force (29.6%) were the common methods for overcoming the victims, who mainly sustained injury around the anogenital region (18.9%). Majority of the anogenital injuries were among victims less than 20 years. All victims except 9.7% received post exposure prophylaxis and all those found eligible for emergency contraception received it (95.0%). Three months follow up was completed by 75.0% of the victims.

The prevalence of sexual assault in our cohort of 0.9% although similar to 0.8% reported by Akinlusi and colleagues from Lagos [1], it was however lower than rates of 2.1-14.0% reported in other parts of Nigeria [3, 4, 10, 11], India [13], Uganda [14] and south Africa [15]. The low rate in our study suggests that recent campaigns are not achieving the expected objectives. However, it may be that lower rate found in our study may be either due to the type of denominator used in our study, the high number of the denominator used or that stigma associated with sexual violence still keeping persons assaulted from reporting. Akinlusi et al [1] similarly adduced large denominator as accountable for their low prevalence. The differential rates as a result of different denominator making comparison difficult, calls for the adoption of a consensus denominator by all. Although it could be argued that the rate is low in our centre, which may warrant the conclusion that sexual assault prevention programmes are working, the increasing trend observed over the years confirms otherwise. The assault prevention activities by government and non-governmental agencies geared towards the prevention of all forms of violence should not only be sustained but intensified. In addition, specific programmes targeting the stigmatisation of sexual assault victims making them not to report for treatment should be developed. The campaigns should emphasise the how the confidentiality of victims are to be maintained. As most Nigerians residing in remote villages may not have the opportunity of access to psychological support, emergency contraception and post exposure prophylaxis, effort should be made to increase access to these services in these remote areas.

Often most report on sexual assault focuses on females, and rightly so as the majority of sexual assault victims are females. However, prevention activities need to start focusing on males too as we found that males were victims of sexual assault in 6.1% of our series. While most reports on sexual assault in Nigeria did not include male victims, studies from India [13], Bangladesh [16], Uganda [14], and South Africa [15] supports our findings that males are also victims of sexual assault. Sexual assault prevention programmes and activities should start addressing factors that facilitate abuse of males especially boys, while intensifying programmes that address sexual assault issues among females. Abused males were comparatively younger than their female counterparts suggesting that these young boys were taken advantage of. Majority (75.0%) of assault on males was through the anal route, pointing to the fact that perpetrators are men that have sex with men. The remaining male victims were forced to have oral sex with the assailant. The society often does not expect males to be sexually assaulted hence parents/guardians leave their wards in the care of potential assailants who soon take advantage of them. Public enlightenment programmes and activities against sexual violence should also include that young boys are also at risk.

The findings of a large percentage of sexual assault victims being persons aged less than 20 years in this study is in keeping with previous studies in Nigeria and elsewhere [1, 3, 4, 10, 11, 13–16]. Adolescent period is a period of adventure and experimentation and they may unknowingly engage in behaviour that exposes them to sexual assault. The findings of most of the assault being perpetrated by persons known to the victims, occurring during the day and in assailants’ homes gives credence to the above statement. In addition being young person they are unlikely to be allowed to go out in the night hence the lower rate of assault during the night time compared to the adults. Also the findings of the majority of assailants being person known to the victims and occurring in assailant homes are similar to previous findings [4, 10–16]. In those studies blood relations, neighbours, acquaintance and authority figures were found to be the main perpetrators like in our study [1, 4, 13–16]. Adolescents and teenagers should therefore be educated on the dangers inherent in total trust of persons known to them especially when such close acquaintances invite them to their homes without their parents’ or guardians’ knowledge. The high number of close acquaintances involved in sexual assault in our setting [3, 4, 10, 11] may also account for the low level of reporting and prosecution of sexual assault perpetrators, as there is the tendency to settle out of court to preserve family respect and maintain friendship.

When the 72 hours window of opportunity to institute prevention activities is taken into consideration, it may be said that our cohort of patients reported on time compared to other studies, as all our patients presented with 96 hours [1, 3, 13, 14]. In these other studies some patients presented for care after 3 weeks to 3 months of assault when neither pregnancy nor infection prevention activities could be instituted. In our study although majority (90.8%) of the victims presented with 72 hours, only 34.6% presented within 24 hours of assault. The import of this is that the very important evidence required for prosecution of the perpetrator is lost. Many reasons have been adduced for this delay ranging from lack of knowledge of where to go for service, limited 24-hours rape crisis centres, effect of alcohol or drugs used to overcome the victims and delay at the police stations [1, 17–19]. In previous studies in Nigeria, sexual assaults were mainly through the vagina [1, 3, 4, 10, 11], in our study a worrisome pattern was noticed as anal assault for both male and female victims was common. This is however similar to findings in south Africa, India and Bangladesh [13, 15, 16]. The observed new pattern in Nigeria from our study is thus very worrisome as the risk of transmitted infection is much higher through anal intercourse than vaginal intercourse. Even more worrisome is the large number of victims in our study who reported being raped by more than one assailant. Victims reported number of perpetrators ranging from one to seven; with more than 20% having been raped by more than one person. This has implications for both the physical injury sustained as well as the number and load of infection the victims are exposed to.

Adequate care was provided for the victims congruent with their time of presentation. However worrisome is that not all the victims completed their follow up. This made it difficult to determine the success of sexual assault management in this study. Although none of the victims that completed follow up reported pregnancy event or HIV infection, it may be that the event occurred in those who did not complete the follow up visits as they may have been disappointed and thus stopped coming. It is therefore important to institute a good tracking system to follow up these victims not only for determination of treatment success, but for emotional and psychological support as well as determination of reason for default if it occurs. Although our study has contributed to the body of knowledge on the trend and pattern of sexual assault in our setting, it is challenged by a number of issues and thus the result may be generalised with caution. Our study was institutional-based and within a HIV treatment programme. This may have affected the prevalence thus may not be a true reflection at population level. However the increasing trend and changing pattern of sexual assault was informative as it is not prone to bias from the system or the setting. The study design is also retrospective and prone to loss of important data points. This study was conducted in a research Institute and data collected by trained researchers using routine based data capture instruments that ensured that all data points are collected during the care of sexual assault victims.

This study generated some important findings that are not readily available in our setting ranging from the increased trend, involvement of male victims and sexual assault treatment success. Information obtained will feed into sexual assault prevention programmes and activities in our setting and elsewhere.

## Conclusion

Sexual assault is common in our setting and its prevalence is increasing with changing pattern. Males are increasingly been assaulted, but female victims are still in the majority. Young persons less than 20 years constituted majority of the victims with persons known to them being the perpetrators in most cases. Although majority of the victims presented within 72 hours of assault, some others presented beyond 72hours when infection transmission and pregnancy could not be prevented. It is recommended that the current effort of public education and enlightenment on the evils of sexual violence and the need for the victims to present early should be intensified. Special programmes targeting young women and men on how to prevent behaviours that put them at risk of sexual violence should be introduced in schools.

### What is known about this topic

- Sexual violence against women is common;
- Sexual violence perpetrated by person known to the victims.

### What this study adds

- Increasing trend of sexual violence in south-western Nigeria;
- Changing pattern with a large number of perpetrators not known to the victims;
- Male victims increasingly been involved.
